# The impact of maternal protein restriction during rat pregnancy upon renal expression of angiotensin receptors and vasopressin-related aquaporins

**DOI:** 10.1186/1477-7827-8-105

**Published:** 2010-08-31

**Authors:** Ruth Cornock, Simon C Langley-Evans, Ali Mobasheri, Sarah McMullen

**Affiliations:** 1 School of Biosciences, University of Nottingham, Sutton Bonington Campus, Loughborough, LE12 5RD, UK; 2 School of Veterinary Medicine and Science, University of Nottingham, Sutton Bonington Campus, Loughborough, LE12 5RD, UK

## Abstract

**Background:**

Maternal protein restriction during rat pregnancy is known to impact upon fetal development, growth and risk of disease in later life. It is of interest to understand how protein undernutrition influences the normal maternal adaptation to pregnancy. Here we investigated the mechanisms regulating renal haemodynamics and plasma volume during pregnancy, in the context of both normal and reduced plasma volume expansion. The study focused on expression of renal angiotensin receptors (ATR) and vasopressin-related aquaporins (AQP), hypothesising that an alteration in the balance of these proteins would be associated with pregnancy *per se *and with compromised plasma volume expansion in rats fed a low-protein diet.

**Methods:**

Female Wistar rats were mated and fed a control (18% casein) or low-protein (9% casein) diet during pregnancy. Animals were anaesthetised on days 5, 10, 15 and 20 of gestation (n = 8/group/time-point) for determination of plasma volume using Evans Blue dye, prior to euthanasia and collection of tissues. Expression of the ATR subtypes and AQP2, 3 and 4 were assessed in maternal kidneys by PCR and western blotting. 24 non-pregnant Wistar rats underwent the same procedure at defined points of the oestrous cycle.

**Results:**

As expected, pregnancy was associated with an increase in blood volume and haemodilution impacted upon red blood cell counts and haemoglobin concentrations. Expression of angiotensin II receptors and aquaporins 2, 3 and 4 was stable across all stages of the oestrus cycle. Interesting patterns of intra-renal protein expression were observed in response to pregnancy, including a significant down-regulation of AQP2. In contrast to previous literature and despite an apparent delay in blood volume expansion in low-protein fed rats, blood volume did not differ significantly between groups of pregnant animals. However, a significant down-regulation of AT_2_R protein expression was observed in low-protein fed animals alongside a decrease in creatinine clearance.

**Conclusion:**

Regulatory systems involved in the pregnancy-induced plasma volume expansion are susceptible to the effects of maternal protein restriction.

## Background

Human pregnancy is associated with a 30-50% increase in plasma volume, beginning early in the first trimester and peaking at around 32 weeks of gestation [1]. Coupled with an expansion of red blood cell mass, this leads to an increase in blood volume [2]. Failure to expand circulating volume is associated with intrauterine growth restriction and hypertensive complications of pregnancy [1,3,4]. A similar profile of plasma volume expansion occurs in rodents during pregnancy [5], providing a useful model with which to examine the molecular and physiological mechanisms of volume expansion and the impact of modifiable environmental factors.

The events leading to plasma volume expansion are not fully understood, but evidence suggests that it is triggered by a fall in systemic vascular tone [6]. This is thought to be followed by compensatory activation of volume-restoring mechanisms, including activation of the renin-angiotensin-aldosterone (RAAS) and arginine-vasopressin (AVP) systems [7,8], leading to renal sodium and water retention. The plasma volume expansion is associated with a substantial increase in effective renal plasma flow (ERPF) and glomerular filtration rate (GFR) from as early as the sixth week of human pregnancy [9,10]. The initial trigger for this series of events remains poorly understood. Activity of the ovaries or corpus luteum is thought to be responsible for the initial peripheral vasodilation, as similar changes are observed in pseudopregnant rats [11] and in women in the luteal phase of the menstrual cycle [12]. A role for the feto-placental compartment has also been suggested, based on fetal reduction experiments in rodents [13] and the greater volume expansion observed in human twin pregnancies [14].

This study aimed to use a rat model of protein restriction to investigate the molecular mechanisms underlying the alterations in renal haemodynamics and plasma volume during pregnancy and to determine the sensitivity of such mechanisms to dietary insult. Low-protein diets are known to decrease urine concentrating ability in humans and rats [15], and are suggested to attenuate plasma volume expansion during pregnancy in rats [16,17]. This is of major interest as offspring from low-protein fed rats develop a range of metabolic disorders in later life, including hypertension, insulin resistance and dyslipidaemia [18,19]. Similar phenotypes have been observed in response to a range of other maternal dietary manipulations. The mechanisms through which such programming of disease risk occurs are not fully understood [20] and the contributions of maternal physiological responses and placental functions have been largely overlooked.

It is possible that inadequate plasma volume expansion may be one mechanism by which maternal diet impacts on the critical developmental processes related to postnatal metabolic disease. Compromised haemodilution and renal adaptation may adversely impact upon placental perfusion and hence the transfer of nutrients to the developing fetus. Inadequate nutrient supply or endocrine signalling may have irreversible effects upon organ development and therefore reduce functional capacity in later life [20]. The current study focused on renal expression of the angiotensin receptors (ATR) and the vasopressin-related aquaporins (AQP) during the oestrous cycle and pregnancy. It has been suggested that activation of arterial baroreceptors in response to peripheral vasodilation leads to nonosmotic AVP release and activation of the RAAS, over-riding the suppression of AVP release which would normally be observed in a hypo-osmotic state and thus contributing to water and sodium retention during pregnancy [21]. It has also been shown that the reduction in urine concentrating ability observed in low-protein fed rats is associated with a decrease in AQP2 protein in the inner medullary tip [15]. We therefore hypothesised that an alteration in the balance of ATR subtypes and the expression of renal AQPs would be observed in the pregnant versus non-pregnant state, and would result in a reduced plasma volume expansion in low-protein fed rats.

## Methods

### Animal procedures

All experiments were carried out in accordance with the 1986 Animals (Scientific Procedures) Act. 64 virgin female Wistar rats (Harlan Ltd, UK) were mated at weights of 200-250 g. Upon confirmation of mating by the presence of a semen plug on the cage floor, rats were assigned to either a control diet (180 g casein/kg) or a low-protein diet (90 g casein/kg) as described previously [22]. Within each dietary group, creatinine clearance and plasma volume were estimated at days 5, 10, 15 and 20 of gestational age (GA, *n *= 7-9 in each dietary GA group) prior to euthanasia. A further group of non-pregnant virgin female Wistar rats (n = 24) were maintained on a standard laboratory chow diet (Harlan Ltd) and their stage of oestrous was determined daily by vaginal swabbing. According to microscopic evaluation of the cell types present, non-pregnant rats were classified as pro-oestrous, oestrous, met-oestrous and di-oestrous, and 6 animals were euthanased per stage of oestrous following estimation of creatinine clearance and plasma volume. Following euthanasia of pregnant and non-pregnant animals, one kidney was snap frozen and stored at -80°C for molecular analyses.

### Creatinine clearance

On the day prior to euthanasia, animals were housed in metabolism cages for 24 hour collection of urine. UK Home Office restrictions did not allow animals to be housed in metabolism cages for more than 24 hours. An aliquot of urine was frozen at -20°C until analysed. Plasma and urine creatinine concentrations were determined by the Jaffé alkaline picrate method [23]. A standard curve was prepared using commercially available creatinine standards (Sigma, UK). A working reagent was created at the time of sample analysis by mixing equal volumes of three solutions: A (4.4 g sodium hydroxide, 9.5 g trisodium phosphate, 9.5 g sodium tetraborate; in 400 mls distilled water), B (4% (w/v) sodium dodecyl sulphate) and C (picric acid). 200 μl of working reagent was added to 20 μl aliquots of samples and standards on a microplate in duplicate. Following 30 minutes incubation at room temperature on an orbital shaker, the absorbance was read at 492 nm. 10 μl of 30% (v/v) acetic acid was then added to each well, the microplate incubated at room temperature for a further five minutes and the absorbance read at 492 nm. The difference between absorbance before and after acidification by acetic acid was calculated. Creatinine clearance was estimated as (urinary creatinine [μmol/l] × volume urine produce in 24 hours [mls])/(plasma creatinine [μmol/l] × 1440 [minutes]). The intra- and inter-assay coefficients of variation for plasma creatinine were 3.1% and 5.5% respectively. The intra and inter-assay coefficients of variation for urinary creatinine were 3.5% and 0.5% respectively.

### Blood and plasma volume

The method for determining blood and plasma volume was based on a previously published method [24]. Under isofluorane anaesthesia, a cannula was inserted into the left iliac vein through which an initial (baseline) blood sample of 1 ml was taken for use as a plasma blank and for analyses of plasma creatinine and haematological parameters. 0.3 ml Evans Blue Dye (0.5 mg/ml) was injected via the cannula, followed by a flush with 0.5 ml saline. The dye was allowed to circulate for five minutes, after which a final blood sample was taken. The animal was then euthanased by injection of sodium pentobarbitone, with death confirmed by cervical dislocation. Blood was collected into EDTA microtubes and centrifuged at 3000 rpm for collection of plasma, which was stored at -20°C prior to analyses.

75 μl of the baseline and final plasma samples were added in duplicate to a 96 well microplate. Plates were read immediately at 620 nm (Tecan Sunrise, Magellan Software version 4.0) and the absorbance of the baseline plasma samples was subtracted from the absorbance of the final plasma samples collected 5 minutes after dye injection. Baseline plasma samples from each animal acted as a blank for that individual animal. Plasma volume was calculated as (milligrams dye injected/plasma dye concentration). The inter- and intra-assay coefficients of variation for Evans Blue dye concentrations were 10.0% and 4.3% respectively. Blood volume was calculated as (plasma volume)/(1-(0.009 × haematocrit)) using an F-cells ratio of 0.9 to account for the difference between whole body and venous haematocrit (Blair & Mickelsen 2006).

### Haematology analyses

Haematological parameters were measured in an aliquot of the baseline blood sample using the Vet Medonic CA 620 (Boule Medical, Sweden) within three hours of collection from the animal.

### Western blotting

Tissues were homogenised in an extraction buffer containing 50 mM Tris/HCL and 5 mM EDTA. Protein concentration was determined by the Bradford method [25] and samples adjusted to equal concentrations. Samples were diluted with an equal volume of loading buffer [4% (w/v) SDS, 125 mM Tris/HCl pH 6.8, 20% (v/v, 87%) glycerol, 0.1 M dithiothreitol] and heated at 90°C for 5 minutes before being run on SDS-polyacrylamide gels. Electrophoresis was carried out in a 10× Tris/glycine/SDS running buffer (National Diagnostics, USA). Following separation by electrophoresis, proteins were transferred to nitrocellulose membrane (GE Healthcare, UK). Blots were probed with the following anti-rat antibodies: AT_1_R diluted 1:500 (Santa Cruz, USA), AQP2[26], AQP3 [27] and AQP4 [27], diluted 1:5000 (affinity purified rabbit anti-rat AQP2 antibodies, was kindly provided by Dr. David Marples, University of Leeds), AT_2_R diluted 1:45,000 (Abcam, UK) and tubulin diluted 1:30,000 (Abcam). The AT_1_R antibody did not distinguish between AT_1a_R and AT_1b_R isoforms. Blots were then treated with goat anti-rabbit horseradish peroxidise linked secondary antibody (GE Healthcare). Blots treated with AQP2 and AT_1_R antibodies were developed using Enhanced Chemiluminescence (ECL, Biological Industries, Israel). Blots treated with AT_2_R and tubulin were developed using ECL Advance (GE Healthcare). Blots were exposed to Hyperfilm ECL (GE Healthcare) to visualise the protein bands, which were quantified using a Quantity-One Multi Analyst system (Bio-Rad, UK). Protein expression was normalised to tubulin expression to correct for any discrepancies in the loading of samples onto the gel.

### RNA extraction and real-time RT-PCR

RNA was extracted from snap-frozen kidney tissue by the TRIzol procedure (Invitrogen, UK) and subjected to DNAse treatment (Promega, UK), phenol-chloroform extraction and ethanol precipitation. RNA was reverse transcribed using Moloney murine leukemia virus (MMLV) reverse transcriptase (Promega). Real-time PCR primers and a probe were designed for AQP2 using Primer Express software (version 1.5; Applied Biosystems) from the DNA sequence GenBank Accession no. NM_012909. The primer sequences were as follows: AQP2 forward primer 5'-CCATTGGTTTCTCTGTTACCCTG-3', reverse primer 5'-CGGGCTGGCTTCATGGAG-3', probe 5'-CCACCTCCTTGGGATCTATTTCACCGG-3'. Primers were ordered from MWG Biotech, Germany. Primer and probe sequences for AT_1a_R, AT_2_R and β-actin are published elsewhere [28]. Real Time PCR was performed using a Lightcycler 480 PCR machine (Roche, UK).

### Statistical analysis

Data is presented throughout as means ± standard error of the mean (SEM). Data was analysed using SPSS version 16.0. To assess the effect of pregnancy *per se*, an independent t-test was used to compare means between non-pregnant and pregnant control fed animals, using all data from each condition irrespective of gestational age or stage of oestrus. In the pregnancy data sets, the effects of gestational age and diet during pregnancy were assessed by two-way analysis of variance. In the figures and tables, superscript letters are used to indicate outcomes of post hoc tests (Bonferroni) applied where ANOVA showed a main effect of diet or gestational age. Posthoc tests cannot be performed on interactions of these factors and so no symbols are shown where only interactive effects were noted. The statistical significance of the main factors and the interaction between them are presented throughout. In the oestrous cycle data sets, the effect of stage of oestrous was assessed by one-way analysis of variance. A probability of <5% was considered statistically significant.

## Results

### Weight, haematological parameters and pregnancy outcome

At the start of the experiment the two dietary groups were of similar weight (control 240 ± 5 g, low protein 247 ± 5 g, not significantly different). There was no significant effect of a maternal low-protein diet on maternal body weight or pregnancy weight gain at any stage of gestation (Table 1). There was a small but statistically significant increase in maternal kidney weight as pregnancy progressed in both the control and low-protein fed groups (Table 1, GA: *P *< 0.05). Significant fluctuations in kidney weight were also observed in non-pregnant rats during the oestrous cycle (Table 2, *P *< 0.05). Urine output was highly variable among pregnant rats but was not significantly influenced by gestational age or diet (Table 1). Non-pregnant animals (Table 2) produced less urine than pregnant animals (P = 0.031), but urine volume was not influenced by stage of oestrus cycle.

**Table 1:** Maternal weight and haematological parameters and litter characteristics at days 5, 10, 15 and 20 of gestation in rats fed a control or low-protein diet from mating.

	Diet	Gestational age (n = 7-9)				Statistical significance		
		5	10	15	20	GA	Diet	GA* Diet
Body weight (g)	Control	257.3 ± 10.7^d^	312.8 ± 6.2^c^	325.3 ± 12.1^b^	372.8 ± 15.0^a^	P < 0.001	NS	NS
	Low protein	277.8 ± 8.2^d^	302.6 ± 10.7^c^	347.3 ± 7.4^b^	346.7 ± 12.8^a^			
Pregnancy weight gain (g)	Control	26.2 ± 2.0^d^	63.8 ± 6.8^c^	82.7 ± 7.2^b^	137.3 ± 7.9^a^	P < 0.05	NS	NS
	Low protein	19.8 ± 3.0^d^	60.4 ± 6.3^c^	90.2 ± 5.2^b^	122.7 ± 13.2^a^			
Kidney weight (g)	Control	1.04 ± 0.04^b^	1.10 ± 0.04	1.10 ± 0.04^a^	1.11 ± 0.06	P < 0.05	NS	NS
	Low protein	0.88 ± 0.11^b^	1.11 ± 0.05	1.14 ± 0.04^a^	0.97 ± 0.05			
Urine volume (ml/24 hour)	Control	18.3 ± 3.5	26.09 ± 4.7	22.4 ± 5.6	29.5 ± 7.4	NS	NS	NS
	Low protein	18.2 ± 2.9	26.6 ± 6.7	21.4 ± 3.1	20.5 ± 6.0			
Litter size (pups/litter)	Control			15 ± 1	14 ± 1	NS	NS	NS
	Low protein			14.0 ± 1	14 ± 1			
Mean fetal weight (g)	Control			0.30 ± 0.01	3.66 ± 0.11	NS	NS	NS
	Low protein			0.29 ± 0.01	3.37 ± 0.13			
Mean placental weight (g)	Control			0.22 ± 0.01	0.51 ± 0.03	NS	NS	NS
	Low protein			0.22 ± 0.01	0.53 ± 0.07			
Red blood cells (10^6^/ml)	Control	6.0 ± 0.7	6.3 ± 0.7	6.6 ± 0.3	6.5 ± 0.2	NS	NS	NS
	Low protein	7.4 ± 0.1	6.9 ± 0.1	6.7 ± 0.2	6.3 ± 0.2			
Haemoglobin (g/dL)	Control	11.3 ± 1.3	11.8 ± 1.3	12.4 ± 0.5	11.9 ± 0.4	NS	NS	NS
	Low protein	13.9 ± 0.2	13.0 ± 0.3	12.6 ± 0.2	11.6 ± 0.3			
Haematocrit (%)	Control	30.6 ± 3.6	31.6 ± 3.6	33.3 ± 1.8	32.6 ± 1.2	NS	NS	NS
	Low protein	37.8 ± 0.5	35.3 ± 0.7	33.8 ± 0.5	31.3 ± 0.9			

Data is presented as mean ± SEM. Data was analysed by two-way ANOVA and the statistical significance of main factor and interaction effects is shown (NS - not significant). Superscripts denote the statistical significance of post hoc analysis conducted where there was a significant effect of gestational age (superscript letters indicate a>b>c > d, P < 0.05).

**Table 2:** Weight and haematological parameters in control fed Wistar rats at each stage of the oestrous cycle

	Stage of oestrus cycle (n = 6 per stage)				*P*
	Proestrus	Oestrus	Metestrus	Dioestrus	
Body weight (g)	234.2 ± 8.9	256.5 ± 11.6	233.9 ± 3.7	244.8 ± 9.8	NS
Kidney weight (g)	0.88 ± 0.02^b^	0.92 ± 0.05	0.97 ± 0.02	1.01 ± 0.02^a^	P < 0.05
Urine volume (ml/24 hour)	16.7 ± 3.0	18.4 ± 4.5	18.7 ± 4.7	15.3 ± 1.6	NS
Red blood cells (10^6^/ml)	7.4 ± 0.2	7.3 ± 0.1	7.5 ± 0.2	7.0 ± 0.1	NS
Haemoglobin (g/dL)	14.0 ± 0.5	13.4 ± 0.2	13.7 ± 0.3	13.2 ± 0.2	NS
Haematocrit (%)	37.9 ± 1.3	36.1 ± 0.7	36.9 ± 0.7	35.5 ± 0.8	NS

Data is presented as mean ± SEM. Data was analysed by one-way ANOVA and the statistical significance of the effect of stage of oestrous is shown (NS - not significant). Superscripts denote the statistical significance of posthoc analysis, which were conducted where a significant main factor effect was found (superscript letters indicate a > b, P < 0.05).

There was no effect of maternal diet on litter size or on mean fetal or placental weight at gestational age 15 or 20. Maternal red blood cell count, haemoglobin concentration and haematocrit were all unaffected by gestational age and maternal diet (Table 1). These parameters did not vary according to stage of the oestrous cycle (Table 2). Pregnant animals exhibited significantly decreased maternal red blood cell count (6.6 ± 0.1 vs. 7.2 ± 0.1 10^6^/mm^3^, *P *< 0.05) haemoglobin concentration (12.4 ± 0.2 vs. 13.5 ± 0.2 g/dl, *P *< 0.01) and haematocrit (33.7 ± 0.7 vs. 36.5 ± 0.4%, *P *< 0.05) in comparison to non-pregnant controls.

### Blood volume and creatinine clearance during pregnancy

**Figure 1:**
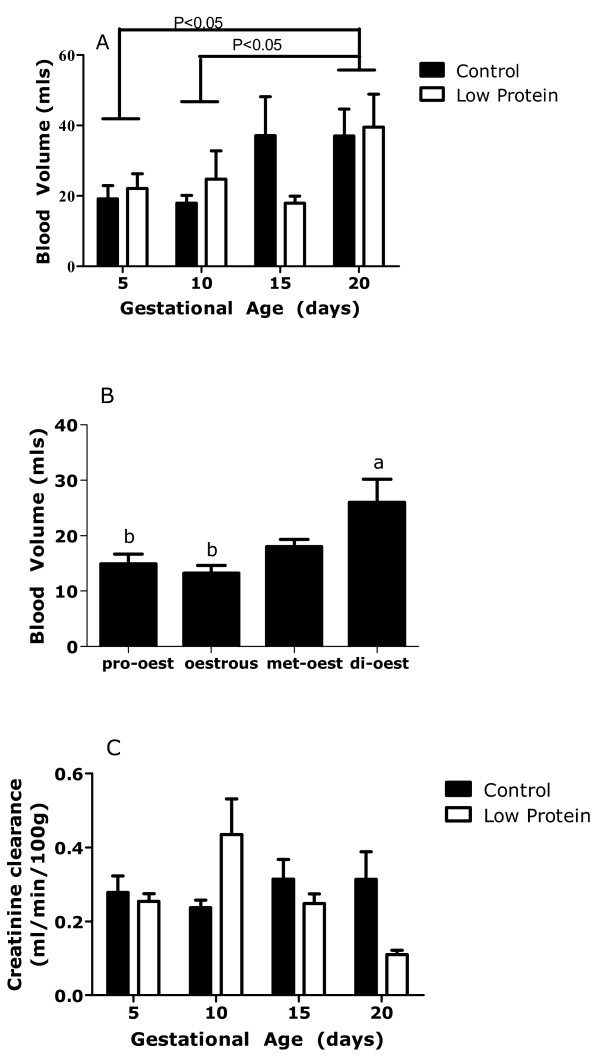
**Blood volume of pregnant and non-pregnant rats**. Blood volume in rats fed control or low protein diet during pregnancy(A, n = 7-9) and in non-pregnant rats at different stages of the oestrus cycle (B, n = 6) Creatinine clearance (C, n = 4-8) in rats fed a control or low-protein diet during pregnancy. Data is presented as mean ± SEM and was analysed by one or two-way ANOVA, as described in the methods section. There was a significant effect of gestational age on blood volume (P < 0.05), but no effect of maternal diet. Blood volume varied with stage of oestrus (superscript letters denote a > b, P < 0.05) There was a significant interaction between gestational age and diet in their effects on creatinine clearance (P < 0.05).

There was a significant effect of gestational age on plasma (data not shown) and blood volume (Figure 1A), with significant expansion of volume being apparent by day 15 in control animals (P = 0.036 compared to non-pregnant animals at oestrus). Although it appeared that there was a delay in the expansion of blood volume in the low-protein fed rats leading to a difference between groups on day 15 of pregnancy, there was no statistically significant interaction between gestational age and diet for either plasma or blood volume. Among the non-pregnant rats there was significant variation in blood volume across the oestrus cycle, with significantly greater volume noted at di-oestrus (Figure 1B).

There was a significant interaction between gestational age and diet in their effects on creatinine clearance during pregnancy (Figure 1C). Whilst clearance remained relatively constant in the control animals, there was a decrease in creatinine clearance in low-protein fed animals between days 10 and 20 of gestation, leading to significantly lower clearance rates on day 20 of gestation.

### Angiotensin receptor expression during pregnancy

**Figure 2:**
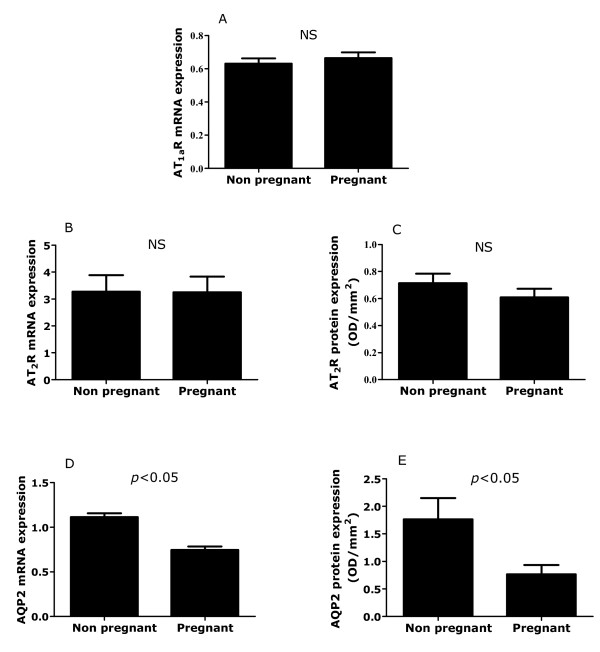
**Expression of angiotensin receptors and aquaporins in pregnant and non-pregnant rats**. Renal expression of AT1R mRNA (A), AT2R (B & C) and AQP2 (D & E) protein and mRNA in non-pregnant (n = 23-24) and pregnant (n = 28-32) rats fed a control diet. Data are presented as mean ± SEM. Data was analysed by independent t-test. AQP2 expression was significantly decreased (P < 0.05) in pregnant versus non-pregnant controls.

**Figure 3:**
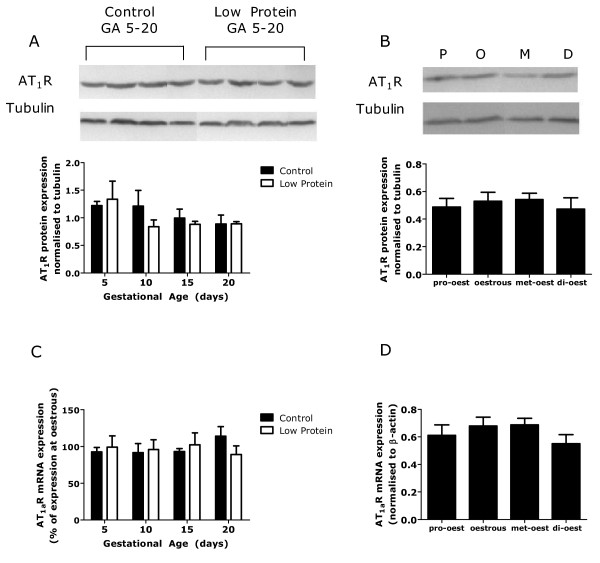
**Expression of renal AT_1_R**. Renal expression of type 1 angiotensin receptor protein and mRNA in kidneys from rats fed a control and low-protein diet during pregnancy (A & C) and in non-pregnant rats at different stages of the oestrus cycle (B & D). Data is presented as mean ± SEM (n = 6-8 per group). Data was analysed by one or two-way ANOVA as described in the methods section. Representative western blots are shown.

There was no difference in the level of AT_1a_R mRNA expression (Figure 2A) between pregnant and non-pregnant rats. It was not possible to compare protein expression between these states due to technical reasons. As pregnancy progressed from GA5 to GA20, there was no alteration in protein or mRNA expression levels and there was no effect of dietary treatment during pregnancy (Figure 3A &3C). Expression of AT_1_R protein and mRNA did not vary across the stages of the oestrus cycle (Figure 3B &3D).

**Figure 4:**
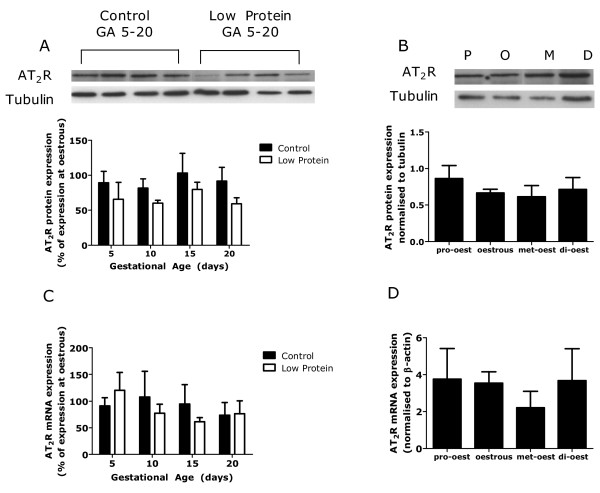
**Expression of renal AT_2_R**. Renal expression of type 2 angiotensin receptor protein and mRNA in kidneys from rats fed a control and low-protein diet during pregnancy (A & C) and in non-pregnant rats at different stages of the oestrus cycle (B & D). Data is presented as mean ± SEM (n = 6-8 per group). Data was analysed by one or two-way ANOVA as described in the methods section. Representative western blots are shown. Protein expression of the AT_2_R was significantly lower in kidneys from low-protein fed rats across all gestational ages. As this was an interaction of gestational age × diet (p < 0.05), no symbols are shown.

The expression of AT_2_R mRNA and protein did not differ significantly between non-pregnant and control fed pregnant animals (Figures 2B, C) and there was no significant effect of gestational age on expression of AT_2_R mRNA or protein (Figure 4A &4C). However, expression of AT_2_R protein was significantly decreased in response to a low-protein diet across all gestational ages (Figure 4A, Diet: *P *< 0.05). Expression of AT_2_R protein and mRNA (Figure 4B &4D) were not significantly influenced by stage of oestrus.

### Expression of AQP2, AQP3 and AQP4 during pregnancy

**Figure 5:**
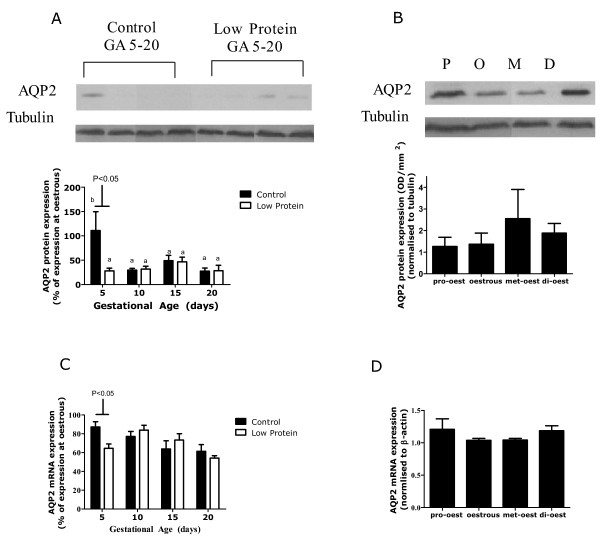
**Expression of renal AQP2**. Renal expression of AQP2 protein and mRNA in kidneys from rats fed a control and low-protein diet during pregnancy (A & C) and in non-pregnant rats at different stages of the oestrus cycle (B & D). Data is presented as mean ± SEM (n = 6-8 per group). Data was analysed by one or two-way ANOVA as described in the methods section. Representative western blots are shown. There was a significant interaction between gestational age and diet in their effects on AQP2 mRNA (P < 0.05) and protein (P < 0.05). Different superscripts show the effects of gestational age on AQP2 expression (b > a, P < 0.05).

**Figure 6:**
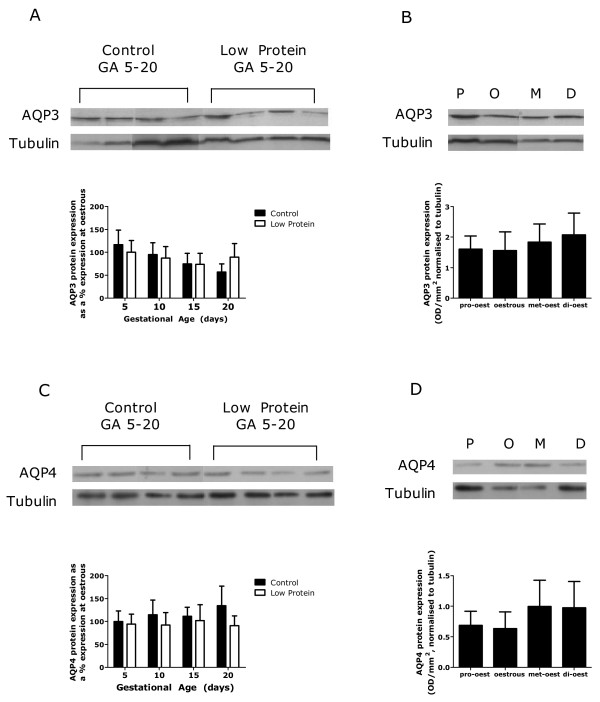
**Expression of renal AQP3 and AQP4**. Renal expression of AQP3 (A & B) and AQP4 (C & D) proteins and mRNA in kidneys from rats fed a control and low-protein diet during pregnancy (A& C) and in non-pregnant rats at different stages of the oestrus cycle (B & D). Data is presented as mean ± SEM (n = 6-8 per group). Data was analysed by one or two-way ANOVA as described in the methods section. Representative western blots are shown.

The expression of AQP2 protein was significantly decreased in pregnancy in comparison to levels at oestrous (Figure 2D, *P *< 0.05). This was associated with a 35% decrease in AQP2 mRNA expression overall (Figure 2E, *P *< 0.05). As pregnancy progressed from GA5 to GA20 there was a significant interaction between gestational age and maternal diet, which reflected an earlier down-regulation of AQP2 in low-protein fed animals in comparison to controls (Figure 5A, GA*Diet *P *< 0.05). There was also an interaction between gestational age and maternal diet in their effects on AQP2 mRNA expression, resulting in a difference between control and low-protein fed animals on GA5 only (Figure 5C, GA*Diet *P *< 0.05). The protein expression of AQP3 and AQP4 did not differ between pregnant and non-pregnant animals (data not shown) and was not affected by gestational age or maternal diet (Figure 6A &6C). The expression of AQP2 protein and mRNA (Figure 5B &5D) and of AQP3 and 4 proteins (Figure 6B &6D) were similar over all stages of the oestrus cycle.

## Discussion

This study used a rat model of protein restriction to investigate the molecular mechanisms regulating renal haemodynamics and plasma volume during pregnancy and the sensitivity of these processes to dietary insult. The study focused on renal expression of the angiotensin receptors and the vasopressin-related aquaporins (AQPs), hypothesising that an alteration in the balance of these proteins would be associated with pregnancy *per se *and with the maternal response to protein restriction.

### Effects of pregnancy per se

In agreement with previous literature, an expansion of plasma and blood volume was observed during pregnancy. Blood volume increased in comparison to that observed in rats at oestrous by 45% at day 5 of pregnancy and 180% at day 20. As expected, the expansion in blood volume was coupled with evidence of haemodilution, as reflected by decreased maternal haematocrit and haemoglobin concentrations

To our knowledge, this is the first study to characterise renal angiotensin receptor protein expression through the course of pregnancy in the rat. No change in AT_1_R or AT_2_R mRNA expression compared to the non-pregnant state was observed and there was no cumulative change in AT_1_R or AT_2_R expression as pregnancy progressed from day 5 to 20. Although a recent paper reported no effect of pregnancy on AT_1_R expression [29], the study focused on mRNA expression only. Previously Bedard *et al*., [30] reported down-regulation of AT_1_R protein expression in the pregnant rat kidney. Observations in non-pregnant animals suggest that binding to AT_1_R is inhibited by 17ß-oestradiol [31]. Progesterone is another endocrine factor that is known to inhibit expression of AT1R and which could have a potent influence during pregnancy [32]. However, in the current study we found that mRNA and protein expression was unchanged in pregnancy. It is important to note that pregnancy-induced plasma volume expansion occurs in the context of systemic and intra-renal vasodilation [33], and an increase in ERPF and GFR [9,10]. This is reflected in the relative systemic pressor resistance to AngII found in pregnancy [34,35]. Although AT_1_R might be envisaged as having effects upon multiple sodium and water transport systems in the nephron, the localization of this receptor is in the proximal tubule. Promotion of sodium reabsorption here is in proportion to GFR and would not impact upon blood volume. Alternatively, although systemic vascular responses to AngII are attenuated, others have shown a normal renal vascular response to AngII [36]. It is possible that site-specific up-regulation of intra-renal AT_1_R may therefore mediate vasoconstriction of the medullary vasculature and contribute to the blunting of the acute pressure natriuresis curve observed in pregnancy [37]. AT_2_R has been suggested to have a role in systemic and renal vasodilation [38] although a role for this receptor in altered renal function during pregnancy has not been fully established. However Ferreira and colleagues observed a 30 fold increase in the mRNA expression of one of the relaxin receptors (LGR7) in the renal cortex. This could result in significant renal vasodilation, allowing glomerular filtration to rise during pregnancy [38]. Relaxin has been suggested to have a major role in both renal and systemic vasodilation in pregnancy [39].

There was a significant down-regulation of the expression of AQP2 protein during pregnancy to 43% of the level observed in non-pregnant animals. AQP2 is the predominant vasopressin-sensitive water channel, cloned and located in the principal cells of the kidney collecting duct [40]. Evidence suggests that the decrease in arterial baroreceptor stretch during pregnancy leads to non-osmotic vasopressin release [21] which activates the vasopressin V_2 _receptors (V_2_Rs) and the adenylate-cyclic AMP pathway in the collecting duct, resulting in increased AQP2 gene transcription and shuttling of AQP2 water channels to the apical membranes of principal cells. Whilst translocation of AQP2 from cytosolic vesicles to the apical membrane mediates the acute response to AVP stimulation [41], long-term regulation of collecting duct water permeability is characterised by an increase in AQP2 mRNA and protein [42,43].

The observed down-regulation of AQP2 mRNA and protein expression during pregnancy is in direct contrast to evidence published previously demonstrating a pregnancy-related up-regulation of AQP2 in renal papillae [44,45]. Segmental differences in aquaporin protein expression in the kidney have been reported by other groups. The utilization of whole kidney samples for analyses was an important limitation of the present study. The increase in AQP2 in pregnancy reported by Ohara *et al*., [44] occurred in the inner medulla and may not be detectable in whole kidney studies. Furthermore, it is possible that translocation of AQP2 from the cytosol to the cell membrane may have interfered with protein extraction and skewed our analyses. It would therefore be more informative to consider the relative distributions of cytosolic and apical AQP2 in future studies and this would be best accomplished through immunohistochemical analysis.

Interestingly, in the study of Ohara *et al*., [44], the up-regulation of AQP2 was observed in the absence of a detectable increase in plasma AVP, but with a role for vasopressin receptor indicated by antagonist studies. Similarly, increased urinary excretion of AQP2 in human pregnancy occurred in the absence of increased circulating AVP concentrations [46]. Given the apparent dissociation between AVP and AQP2 expression in these studies and the contrasting results observed in the current study and that of Ohara et al. [44], further work investigating the regulation of AQP2 expression and shuttling is required before its involvement in the enhanced water reabsorption during pregnancy can be fully understood. AQP3 and AQP4 are both present in the basolateral plasma membrane of collecting duct principal cells [47] and represent exit pathways for water reabsorbed apically via AQP2. The protein expression of these aquaporins remained unaffected by pregnancy *per se*, suggesting that the key regulatory events occur at the apical plasma membrane where the abundance of AQP2 protein creates a "bottleneck" for water reabsorption.

### Effects of a low protein diet

In contrast to previous literature [16,17], feeding a low-protein diet to pregnant rats did not have a statistically significant effect on plasma or blood volume expansion during pregnancy. However, a trend towards delayed volume expansion was noted at day 15 of pregnancy. The study by Rosso & Streeter [16] used a more severe protein restriction (6% versus 25% casein) than that used in the current study (9% versus 18% casein), and the latter may not have been sufficient to impact significantly on plasma volume expansion. However, a previous study using the same experimental dietary formulations as the current study did suggest a significant reduction in plasma volume expansion at day 20 of pregnancy [17]. In the current study, the relative reduction in creatinine clearance and the alterations in expression of key mediators of renal fluid homeostasis in response to a low-protein diet do provide further evidence that the regulatory systems involved in plasma volume expansion are susceptible to maternal protein restriction. However, the impact of the protein restriction was subtle and considerably less than expected. Our findings therefore suggest that maternal adaptation to pregnancy is less sensitive to protein restriction than has been previously inferred [16,17], and that only severe undernutrition has major influence on these processes.

The expression of AT_2_R protein was significantly decreased in low-protein fed rats throughout pregnancy. Enhanced AT_2_R-mediated vascular relaxation pathways have been implicated in the systemic vasodilation of pregnancy, including increased expression and activity of endothelial AT_2_R [48] and involvement in the vasodilation of the uterine artery [49]. As a candidate for mediating counter-regulatory vasodilation in response to AngII in pregnancy, disruption of AT_2_R receptor expression may be involved in inadequate vascular adaptation to pregnancy. Interestingly, a previous study has shown vascular relaxation in response to an endothelium-dependent vasodilator to be impaired in mesenteric arteries of pregnant rats fed a 9% protein diet [50]. This study adds to this previous literature by indicating a role for intra-renal AT_2_R in mediating altered haemodynamic function in pregnant rats fed a low-protein diet. In addition to the alterations in AT_2_R expression, the down-regulation of AQP2 occurred earlier in low-protein fed rats. Given the prevailing evidence of reduced or, as observed here, delayed plasma volume expansion in low-protein-fed dams, we had hypothesised that this would occur and suggest that this signifies a reduced capacity for water reabsorption in low-protein-fed rats.

## Conclusion

To conclude, this study has demonstrated that no change in expression of intrarenal AT_1_R or AT_2_R proteins occurs during pregnancy. Surprisingly, a down-regulation of AQP2 was observed and the role of this water channel during pregnancy remains unclear. A significant down-regulation of AT_2_R protein expression was observed in low protein fed animals alongside a relative decrease in creatinine clearance, providing evidence that regulatory systems involved in plasma volume expansion are susceptible to maternal nutrient restriction. However, the impact of protein restriction on maternal blood volume expansion was relatively minor, suggesting that adaptive responses are able to compensate for variation in nutritional status.

## Abbreviations

AMP: adenosine monophosphate; AQP: aquaporin; AT_1_R: angiotensin II type 1 receptor; AT_2_R: angiotensin II type 2 receptor; AVP: arginine-vasopressin; EDTA: ethylenediaminetetraacetic acid; EPRF: effective renal plasma flow; GA: gestational age; GFR: glomerular filtration rate; RAAS: renin-angiotensin-aldosterone system.

## Competing interests

The authors declare that they have no competing interests.

## Authors' contributions

RC carried out the animal experiments and analyses of gene and protein expression. SLE participated in the design of the study and the drafting of the manuscript. AM contributed to the design of the study and the analyses of AQP expression. SM conceived the study, participated in its design and co-ordination and helped to draft the manuscript. All authors read and approved the final manuscript.

## References

[B1] Salas SP Marshall G Gutierrez B Rosso P Time course of maternal plasma volume and hormonal changes in women with preeclampsia or fetal growth restriction Hypertension 2006 47 203 208 10.1161/01.HYP.0000200042.64517.19 16380519

[B2] Taylor DJ Lind T Red-cell mass during and after normal-pregnancy BJOG 1979 86 364 370 10.1111/j.1471-0528.1979.tb10611.x 465384

[B3] Duvekot JJ Cheriex EC Pieters FAA Menheere P Schouten HJA Peeters LLH Maternal volume homeostasis in early-pregnancy in relation to fetal growth restriction Obstet Gynecol 1995 85 361 367 10.1016/0029-7844(94)00417-C 7862373

[B4] Salas SP Rosso P Plasma volume, renal function, and hormonal levels in pregnant women with idiopathic fetal growth restriction or preeclampsia Hypertens Pregnancy 1998 17 69 79 10.3109/10641959809072239

[B5] Atherton JC Dark JM Garland HO Morgan MRA Pidgeon J Soni S Changes in water and electrolyte balance, plasma volume and composition during pregnancy in the rat J Physiol 1982 330 81 93 7175756 10.1113/jphysiol.1982.sp014330 PMC1225287

[B6] Duvekot JJ Cheriex EC Pieters FAA Menheere P Peeters LLH Early-pregnancy changes in hemodynamics and volume homeostasis are consecutive adjustments triggered by a primary fall in systemic vascular tone Am J Obstet Gynecol 1993 169 1382 1392 8267033 10.1016/0002-9378(93)90405-8

[B7] Al Kadi H Nasrat H Pipkin FB A prospective, longitudinal study of the renin-angiotensin system, prostacyclin and thromboxane in the first trimester of normal human pregnancy: association with birthweight Human Reprod 2005 20 3157 3162 10.1093/humrep/dei184 16006463

[B8] Schrier RW Cadnapaphornchai MA Ohara M Water retention and aquaporins in heart failure, liver disease and pregnancy J Royal Soc Med 2001 94 265 269 10.1177/014107680109400603 PMC1281519 11387413

[B9] Chapman AB Abraham WT Zamudio S Coffin C Merouani A Young D Johnson A Osorio F Goldberg C Moore LG Dahms T Schrier RW Temporal relationships between hormonal and hemodynamic changes in early human pregnancy Kid Intl 1998 54 2056 2063 10.1046/j.1523-1755.1998.00217.x 9853271

[B10] Bekheirnia MR Schrier RW Pathophysiology of water and sodium retention: edematous states with normal kidney function Curr Op Pharmacol 2006 6 202 207 10.1016/j.coph.2005.09.008 16483846

[B11] Paller MS Gregorini G Ferris TF Pressor responsiveness in pseudopregnant and pregnant rats - role of maternal factors Am J Physiol 1989 257 R866 R871 2802003 10.1152/ajpregu.1989.257.4.R866

[B12] Chapman AB Zamudio S Woodmansee W Merouani A Osorio F Johnson A Moore LG Dahms T Coffin C Abraham WT Schrier RW Systemic and renal hemodynamic changes in the luteal phase of the menstrual cycle mimic early pregnancy Am J Physiol 1997 273 F777 F782 9374841 10.1152/ajprenal.1997.273.5.F777

[B13] Van Mieghem T van Bree R Van Herck E Deprest J Verhaeghe J Insulin-like growth factor-II regulates maternal hemodynamic adaptation to pregnancy in rats Am J Physiol 2009 297 R1615 R1621 10.1152/ajpregu.00463.2009 PMC2777779 19776249

[B14] Hytten F Blood-volume changes in normal-pregnancy Clin Haematol 1985 14 601 612 4075604

[B15] Sands JM Naruse M Jacobs JD Wilcox JN Klein JD Changes in aquaporin-2 protein contribute to the urine concentrating defect in rats fed a low-protein diet J Clin Invest 1996 97 2807 2814 10.1172/JCI118736 8675692 PMC507374

[B16] Rosso P Streeter MR Effects of food or protein restriction on plasma-volume expansion in pregnant rats J Nutr 1979 109 1887 1892 501438 10.1093/jn/109.11.1887

[B17] Welham SJM Wheeler T Langley-Evans SC Maternal plasma volume expansion is modulated in early pregnancy by a low protein diet in the rat Clin Sci 1998 94 M15

[B18] McMullen S Mostyn A Animal models for the study of the developmental origins of health and disease Proc Nutr Soc 2009 68 306 320 10.1017/S0029665109001396 19490740

[B19] Langley-Evans SC Jackson AA Intrauterine programming of hypertension: nutrient-hormone interactions Nutr Reviews 1996 54 163 169 10.1111/j.1753-4887.1996.tb03923.x 8810822

[B20] Langley-Evans SC McMullen S Developmental origins of adult disease Med Princ Pract 2010 19 87 98 10.1159/000273066 20134170

[B21] Schrier RW Water and sodium retention in edematous disorders: Role of vasopressin and aldosterone Am J Med 2006 119 S47 S53 10.1016/j.amjmed.2006.05.007 16843085

[B22] Langley-Evans SC Gardner DS Jackson AA Association of disproportionate growth of fetal rats in late gestation with raised systolic blood pressure in later life J Reprod Fertil 1996 106 307 312 10.1530/jrf.0.1060307 8699415

[B23] Bowers LD Wong ET Kinetic serum creatinine assays. 2. A critical-evaluation and review Clin Chem 1980 26 555 561 7020989

[B24] Blair ML Mickelsen D Plasma protein and blood volume restitution after hemorrhage in conscious pregnant and ovarian steroid-replaced rats Am J Physiol 2006 290 R425 R434 10.1152/ajpregu.00011.2005 16166212

[B25] Bradford MM Rapid and sensitive method for quantitation of microgram quantities of protein utilizing principle of protein-dye binding Anal Biochem 1976 72 248 254 10.1016/0003-2697(76)90527-3 942051

[B26] Mobasheri A Wray S Marples D Distribution of AQP2 and AQP3 water channels in human tissue microarrays J Mol Histol 2005 36 1 14 10.1007/s10735-004-2633-4 15703994

[B27] Floyd RV Mason SL Proudman CJ German AJ Marples D Mobasheri A Expression and nephron segment-specific distribution of major renal aquaporins (AQP1-4) in Equus caballus, the domestic horse Am J Physiol 2007 293 R492 R503 10.1152/ajpregu.00689.2005 17442782

[B28] McMullen S Langley-Evans SC Maternal low-protein diet in rat pregnancy programs blood pressure through sex-specific mechanisms Am J Physiol 2005 288 R85 90 10.1152/ajpregu.00435.2004 15374820

[B29] Ferreira VM Gomes TS Reis LA Ferreira AT Razvickas CV Schor N Boim MA Receptor-induced dilatation in the systemic and intrarenal adaptation to pregnancy in rats Plos One 2009 4 e4845 10.1371/journal.pone.0004845 19287481 PMC2653634

[B30] Bedard S Sicotte B St-Louis J Brochu M Modulation of body fluids and angiotensin II receptors in a rat model of intra-uterine growth restriction J Physiol 2005 562 937 950 10.1113/jphysiol.2004.064683 15539403 PMC1665548

[B31] Rogers JL Mitchell AR Maric C Sandberg K Myers A Mulroney SE Effect of sex hormones on renal estrogen and angiotensin type 1 receptors in female and male rats Am J Physiol 2007 292 R794 R799 10.1152/ajpregu.00424.2006 16990489

[B32] Kalenga MK De Gasparo M Thomas K De Hertogh R Down-regulation of angiotensin AT1 receptor by progesterone in human placenta J Clin Endocrinol Metab 1996 81 998 1002 10.1210/jc.81.3.998 8772564

[B33] Conrad KP Mechanisms of renal vasodilation and hyperfiltration during pregnancy J Soc Gynecol Invest 2004 11 438 448 10.1016/j.jsgi.2004.05.002 15458740

[B34] Gant NF Daley GL Chand S Whalley PJ Macdonal PC Study of angiotensin-II pressor response throughout primigravid pregnancy J Clin Invest 1973 52 2682 2689 10.1172/JCI107462 4355997 PMC302534

[B35] Baker PN Pipkin FB Symonds EM Longitudinal-study of platelet angiotensin-II binding in human-pregnancy Clin Sci 1992 82 377 381 1315647 10.1042/cs0820377

[B36] Masilamani S Baylis C The renal vasculature does not participate in the peripheral refractoriness to administered angiotensin II (AII) in the late pregnant (LP) rat J Am Soc Nephrol 1992 3 566

[B37] Masilamani S Hobbs GR Baylis C The acute pressure natriuresis response blunted and the blood pressure response reset in the normal pregnant rat Am J Obstet Gynecol 1998 179 486 491 10.1016/S0002-9378(98)70384-9 9731858

[B38] Ferreira VM Gomes TS Reis LA Ferreira AT Razvickas CV Schor N Boim MA Receptor-induced dilatation in the systemic and intrarenal adaptation to pregnancy in rats Plos One 2009 4 e4845 10.1371/journal.pone.0004845 19287481 PMC2653634

[B39] Debrah DO Novak J Matthews JE Ramirez RJ Shroff SG Conrad KP Relaxin is essential for systemic vasodilation and increased global arterial compliance during early pregnancy in conscious rats Endocrinology 2006 147 5126 5131 10.1210/en.2006-0567 16873529

[B40] Fushimi K Uchida S Hara Y Hirata Y Marumo F Sasaki S Cloning and expression of apical membrane water channel of rat-kidney collecting tubule Nature 1993 361 549 552 10.1038/361549a0 8429910

[B41] Nielsen S Chou CL Marples D Christensen EI Kishore BK Knepper MA Vasopressin increases water permeability of kidney collecting duct by inducing translocation of aquaporin-CD water channels to plasma-membrane Proc Natl Acad Sci USA 1995 92 1013 1017 10.1073/pnas.92.4.1013 7532304 PMC42627

[B42] Yamamoto T Sasaki S Fushimi K Kawasaki K Yaoita E Oota K Hirata Y Marumo F Kihara I Localization and expression of a collecting duct water channel, aquaporin, in hydrated and dehydrated rats Exp Nephrol 1995 3 193 201 7542539

[B43] Saito T Ishikawa SE Sasaki S Fujita N Fushimi K Okada K Takeuchi K Sakamoto A Ookawara S Kaneko T Marumo F Alteration in water channel AQP-2 by removal of AVP stimulation in collecting duct cells of dehydrated rats Am J Physiol 1997 272 F183 F191 9124394 10.1152/ajprenal.1997.272.2.F183

[B44] Ohara M Martin PY Xu DL St John J Pattison TA Kim JK Schrier RW Upregulation of aquaporin 2 water channel expression in pregnant rats J Clin Invest 1998 101 1076 1083 10.1172/JCI649 9486978 PMC508659

[B45] Abreu N Tardin JC Boim MA Campos RR Bergamaschi CT Schor N Hemodynamic parameters during normal and hypertensive pregnancy in rats:evaluation of renal salt and water transporters Hypertens Pregnancy 2008 49 63 18293204 10.1080/10641950701825887

[B46] Buemi M D'Anna R Di Pasquale G Floccari F Ruello A Aloisi C Leonardi I Frisina N Corica F Urinary excretion of aquaporin-2 water channel during pregnancy Cell Physiol Biochem 2001 11 203 208 10.1159/000047807 11509828

[B47] Nielsen S Frokiaer J Marples D Kwon TH Agre P Knepper MA Aquaporins in the kidney:From molecules to medicine Physiol Rev 2002 82 205 244 11773613 10.1152/physrev.00024.2001

[B48] Stennett AK Qiao XY Falone AE Koledova VV Khalil RA Increased vascular angiotensin type 2 receptor expression and NOS-mediated mechanisms of vascular relaxation in pregnant rats Am J Physiol 2009 296 H745 H755 10.1152/ajpheart.00861.2008 PMC2660233 19151255

[B49] Hannan RE Davis EA Widdop RE Functional role of angiotensin II AT(2) receptor in modulation of AT(1) receptor-mediated contraction in rat uterine artery:involvement of bradykinin and nitric oxide Br J Pharmacol 2003 140 987 995 10.1038/sj.bjp.0705484 14530222 PMC1574089

[B50] Koumentaki A Anthony F Poston L Wheeler T Low-protein diet impairs vascular relaxation in virgin and pregnant rats Clin Sci 2002 102 553 560 10.1042/CS20010252 11980575

